# The Treatment of Corneal Ulcers

**Published:** 1911-01-28

**Authors:** 


### Resident Medical Officers' Department.

### THE TREATMENT OF CORNEAL ULCERS.

A large proportion (10 per cent.) of eye in-patients are admitted for corneal ulceration. The majority of these are traumatic in origin and are produced in miners and stoneworkers by sharp particles of coal or stone striking the cornea or getting into the conjunctival sac. This type of ulcer is fairly deep, quickly growing, situated in the lower two-thirds of the cornea; it presents a well-defined, opaque, crescentic border, the hollow of the crescent looking towards the middle line of the cornea. The advancing border is densely infiltrated with leucocytes, and the entire ulcerated area is less transparent than the normal cornea.

### The Cause of Corneal Ulcers.

Almost 50 per cent. of these cases suffer from chronic conjunctivitis and regurgitation of muco-pus from their lachrymal sacs; as bacteriological examination of similar particles of stone and coal is negative it is more than probable that the offending organisms exist in the conjunctival sac before the accident, which removes the protective epithelium.

The associated iritis, as well as the exudate into the anterior chamber, are directly proportionate to the severity of the ulceration and the virulence of the infection. Corneal abscesses, collections of pus cells in the layers of the cornea, are serious complications.

### Treatment in Simple Cases.

If seen early enough when the ulcer is just below Bowman's membrane and before the crescentic border has appeared, it is sufficient to douche the conjunctival sac with warm saline or boracic lotion, and to touch the ulcer with pure carbolic acid. This is best applied with the end of a fine probe, round which a small piece of wool has been firmly twisted. When the sharpened end of a wooden match is used the acid is apt to run over the cornea, and it is not uncommon to find on the following day fresh points of infection due to abrasions of the epithelium. The acid on the wool-covered probe, even if it be saturated, is limited to the spot touched, and there is no possibility of damage to the epithelium.

### Advanced Cases.

When the advancing edge has appeared, touching with carbolic acid is too uncertain; best results are obtained by staining the ulcer with 2 per cent. fluorescine, after anæsthetising the conjunctival sac with 5 per cent. cocaine, and then by applying the galvano-cautery. If there is much congestion a drop of adrenalin chloride (1 in 1000) increases the action of the cocaine. The cautery at a dull red heat is applied to the whole of the stained area, and just beyond it in the region of the crescentic border. Atropine sulphate drops (1 per cent.) are instilled three or four times daily, and hot fomentations applied two-hourly during the day and less often at night. The patient should be kept in bed.

If ocular pain and ciliary congestion are marked four to eight leeches applied to the corresponding temple will give great relief. The addition of one drachm of the green extract of belladonna to each fomentation will also be beneficial. Frontal headache is best treated by small doses (gr. j.) of hydrarg. subchlor. four hourly, combined with 5 gr. of aspirin.

If the above treatment is not sufficient to cause the disappearance of the hypopyon in thirty-six to forty-eight hours, or should fresh points of infection appear around the cauterised area, Sämisch's section must be made through the ulcer. A general or local anæsthetic may be given.

### Douching with Mercury.

The lids are kept apart by a speculum and the conjunctiva and cornea thoroughly douched with biniodide of mercury lotion (1 in 6000). After the douching the globe is steadied with a pair of fixation forceps, and a Graefe knife is made to transfix the cornea. Entering the anterior chamber through healthy cornea, the counter-puncture at the opposite side is also made through healthy cornea; the blade of the knife cuts outwards through the base of the ulcer. Pure carbolic acid may be applied to the ulcer before the section. This operation is preferable to a second cauterisation of the ulcer, and is much more effective than peripheral incisions or punctures with a keratome or broad needle. Prolapse of the iris very rarely occurs.

### Results of Treatment.

Ulcers treated after this manner heal with very little scarring, and the line of incision is hardly visible afterwards; a second application of the galvano-cautery, on the other hand, leaves a dense white scar, or the base of the ulcer is so thinned that Descemet's membrane protrudes, and anterior staphyloma results sooner or later. Healing is slower, too, after perforation with the cautery.

### Abscesses and Complications.

Corneal abscesses should be opened with the Graefe knife. In patients suffering from dacryocystitis re-infection frequently occurs after cauterisation of the ulcer. There is a marked improvement for the first twenty-four hours. On the second day the hypopyon increases, and iritis is marked. The ulcer is covered with a greyish slough and the cornea around it is infiltrated. To prevent this the canaliculi must be obliterated just before cauterising the ulcer.

They are dilated, the contents of the sac well pressed out, and the point of the galvano-cautery inserted before the current is turned on. Should the sac get very tense in two or three days, some of the contents may be drawn off with a hypodermic syringe, or a small incision may be made down to the sac.

Care must be taken to prevent the pus from getting into the conjunctival sac, although by this time the ulcer is clean and healing rapidly.

The non-traumatic ulcers are either marginal or central. The former are deep and limited, and the latter superficial and spreading. General treatment is more important than local. This also applies to the superficial ulcerations in children.

Accompanying conjunctivitis should be treated with mild astringents. The following silver preparations are useful in the order given: Sophol, protargol, argyrol.

After the ulcer has healed a great deal can be done to lessen the subsequent scarring and increase the transparency of the cornea by the use of certain irritants such as hydrarg. subchlor, hydrarg. oxid. flav., etc. With the help of the corneal trephine, too, circles of transparent cornea at the periphery can be exchanged for central opaque circles.

